# Citizen Science and Multimedia Didactic Resources: Knowledge of Mosquito-Borne Diseases in an Urban Area of Southwestern Colombia

**DOI:** 10.3390/tropicalmed9110256

**Published:** 2024-10-25

**Authors:** Francisco Javier Bedoya-Rodríguez, Carlos Eduardo Guevara-Fletcher, Jonathan S. Pelegrin

**Affiliations:** 1 Research Group in Ecology and Conservation of Biodiversity, Master’s Program in Environmental Education and Sustainable Development and Doctorate in Education, Faculty of Education, Universidad Santiago de Cali, Cali 7600, Colombia; cefletcher8@hotmail.com (C.E.G.-F.); jonathan.pelegrin00@usc.edu.co (J.S.P.); 2 Territorial Dimensions Research Group, Fundación Universitaria de Popayán, Popayán 190003, Colombia; 3 Institute of Marine Sciences and Limnology (INCIMAR), Universidad del Valle, Cali 760042, Colombia

**Keywords:** citizen science, ecological knowledge, environmental education, multimedia teaching strategy, vector-borne diseases

## Abstract

Citizen science resources have had great relevance in community educational intervention, fostering interest in ecological research projects. This study investigated the influence of the application of multimedia didactic resources focused on citizen science and analysis of sociodemographic variables to improve the levels of ecological knowledge about mosquito-borne diseases. For this, a probabilistic sample of 172 participants from an urban sector in southwestern Colombia was selected. A multimedia didactic material was designed for the educational intervention and evaluated by means of pretest and posttest. To assess whether the educational intervention was statistically significant, the data were processed with statistical inference to determine the influence of various variables (gender, age, marital status, schooling, and occupation) on each person’s ecological knowledge. The findings showed a significant increase in the knowledge levels of the participants. The marital status variable (married and cohabiting) significantly influenced ecological knowledge. These participants are more likely to obtain high knowledge, associated with the prevention of their family environment. This study demonstrated that multimedia didactic strategies are an important factor to take into account in the structuring of environmental education and citizen science projects. For future research, it is suggested to deepen the relationship between sociodemographic variables and ecological knowledge.

## 1. Introduction

Worldwide, citizen science is used to strengthen scientific knowledge through the formulation and execution of a structured project of collective, participatory, and open research [1,2]. Applying research in social contexts to understand ecosystems enhances learning and influences social actions and habits. For instance, using digital technologies for biodiversity monitoring boosts scientific knowledge about biodiversity and its connection to humans [3,4,5,6,7,8,9]. Consequently, the community contribution of the rural and/or urban sector involved in citizen science is fundamental to knowing particular ecosystemic and ecological aspects of the sector where they live, since their ancestral and daily knowledge about the environment can provide useful and logistical data to scientists who carry out research projects [3].

Citizen science, through platforms like iNaturalist (a mobile application to identify biodiversity anywhere in the world, www.inaturalist.org, accessed on 15 June 2023), enhances community engagement in biodiversity research by enabling users to document local flora and fauna via mobile observations. This app allows individuals to take photos and contribute to a global database, fostering awareness and interest in their surrounding ecosystems [5,10,11,12]. In addition to the contribution that people make in the citizen science process with the use of these interactive platforms, the study of the motivational factors in their participation in this type of project helps to recognize how the participation of different audiences can be improved [13]. Likewise, the use of this digital application strengthens the scientific spirit, as evidenced by Hitchcock et al. [6]. This study demonstrated how students developed bio-literacy by obtaining and processing ecological data, participating as citizen scientists, and enhancing collaborative learning and scientific knowledge in biodiversity research. In Colombia, Sánchez-Clavijo et al. [14] analyzed the use of digital tools in citizen science during the COVID-19 pandemic, revealing significant interest in contributing to biodiversity research through global platforms.

Citizen science plays a crucial role in studying urban biodiversity, as community involvement enhances research efforts and facilitates the collection and analysis of regional data on urban species [4]. It should be noted that it is becoming increasingly important for researchers, students, and the community in general to obtain and organize biodiversity databases, evidencing the improvement in the quality of the data found and the observations made, which serve as input for different ecological studies [9].

However, citizen science has had a significant impact on both non-formal education (NFE) and formal education, encouraging the public to engage in research and online learning processes that help them understand the natural world [15,16,17]. Likewise, Stanković et al. [18] argued that NFE relies on information and communication technologies (ICTs) to enhance teaching and learning. In Germany, the growing importance of digital media in NFE highlights the need for ongoing educator training in designing these resources [19]. Likewise, the work of Degner et al. [20] considered that digital media contribute significantly to the learning process in non-school environments.

In this sense, multimedia didactic materials also contribute to the environmental education (EE) of the communities, strengthening the teaching of environmental issues, and reinforcing civic commitment [21,22,23,24]. Likewise, the study by Huang et al. [25] concluded that the application of innovative technology motivates learning in education in ecological contexts. However, Tao et al. [26] review noted that digital interactive learning environments have a significant impact on learning. Therefore, citizen science must overcome challenges in partnering with academia to improve research outcomes, prompting educators to enhance their digital skills for the needs of new generations in various educational contexts [27].

Citizen science has contributed to the study of mosquitoes (Diptera: Culicidae) by applying digital epidemiology to prevent infectious diseases transmitted by these vectors; in addition to being a matter of public health and public health interest [28,29]. Likewise, the incursion of smartphones and ICTs in general, to recompile images in real-time and specific locations, favors research on disease vector species and the identification of their potential effects on the human population [8,30,31,32]. In this sense, cell phones have become tools for vector-borne disease (VBD) control and prevention processes [33,34]. Likewise, studies have been developed with digital applications to improve both vector and disease control [35,36,37]. Moreover, in the surveillance of these VBDs, the affected communities have been involved in the design of technologies that benefit their control and prevention [38]. Thus, the success of multimedia tools in the teaching and learning processes has reduced the limitation of access to education [39].

In this sense, citizen science plays a crucial role in dengue prevention, as the active participation of citizens in mosquito data collection fosters both awareness and collective responsibility [40]. This approach is complemented by programs that combine technology and citizen participation, significantly improving the effectiveness of mosquito control through digital platforms [41]. In addition, citizen science provides valuable tools to track mosquitoes and improve epidemiological surveillance, facilitating a more effective response to disease outbreaks [42]. Projects such as Mosquito Alert demonstrate how the integration of citizen and scientific data generates risk maps and early warning systems, strengthening community engagement in public health [43].

Therefore, local knowledge of VBD, the use of digital technologies, and the creation of science networks could facilitate this articulation [44,45]. In turn, citizen science offers a novel and less costly solution to the study of VBD compared to traditional vector monitoring methods [46]. Thus, citizens’ contributions can be integrated into public health education campaigns. For this reason, the purpose of this study is to promote the management of mosquitoes (Diptera: Culicidae) common in urban environments and their potential VBD through a didactic strategy with a citizen science approach in an urban sector of southwestern Colombia, which allows changes in the behavior and ecological knowledge of citizens in a non-formal EE context.

## 2. Materials and Methods

A pretest–posttest methodological design was carried out in a single population group. A pretest was applied to identify initial ecological knowledge [47,48,49,50]. Subsequently, the educational intervention was carried out through the didactic strategy and finally a posttest in order to determine the final level of knowledge. The didactic strategy with a citizen science approach was implemented for eight months, from April 2023 to December 2023. Ethical approval for the study was given by the community action board of the urban sector and the ethics committee of the faculty of education of Universidad Santiago de Cali, who approved the informed consent and the execution of the present exercise.

### 2.1. Study Area and Participants

**Figure 1:**
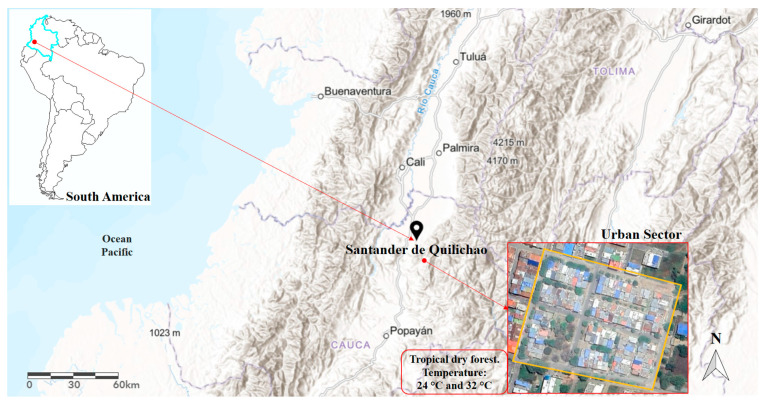
Geographical location of the study sector in the municipality of Santander, Department of Cauca, Colombia, South America.

The present study was conducted in a sector of the urban perimeter of the municipality of Santander de Quilichao (3°00′29.8″ N 76°28′40.1″ W), located in the department of Cauca, southwestern Colombia (Figure 1). This region has a humid tropical climate, with dry and rainy climatic seasons and a temperature that ranges between 24 °C and 32 °C [51], these conditions classify this region bioclimatically as a tropical dry forest, whose conditions favor the proliferation of mosquitoes and, therefore, VBD.

A probabilistic random selection (95% confidence) of 172 participants, out of a total population of 310, was made for the application of the test. The design of the multimedia material took into account information on the ecology of mosquitoes (Diptera: Culicidae) and information on VBD in the sector to be evaluated. When choosing the type of material, the sociodemographic characterization was taken into account, as well as being able to identify that the participants have access to cell phones with multimedia visualization. The distribution of the participants is mostly female, with 57% women and 43% men. Most of the participants are in the age range of 28–37 years (32.6%) and 38–47 years (32.6%), while the smallest group corresponds to people aged 48 years or older (15.1%). In terms of marital status, there is a predominance of singles (37.8%) and married people (32.6%). Schooling is notably high, with 59.3% of respondents having a university education. In terms of occupation, the majority are employed (57%), followed by self-employed (29.1%) (Appendix A).

### 2.2. Pretest, Educational Intervention, and Posttest

To examine the effect of the strategy, five validated questions were adapted and used in surveys by the World Health Organization [52]: How are mosquitoes formed?; Which people can contract dengue, Zika, chikungunya, malaria, or other such viruses?; How can a person contract VBD?; What are the signs and symptoms of dengue virus disease?; Is there a treatment for VBD infection? An initial pretest assessment was conducted, with scores from 0 to 5, to determine the level of knowledge about VTE and culicids. The ratings were defined as follows: 0 = no knowledge, 1 = very low knowledge, 2 = low knowledge, 3 = medium knowledge, 4 = high knowledge, and 5 = very high knowledge. The same person was responsible for all ratings, thus ensuring consistency in the evaluation. A standardized set of questions assessing specific aspects of VBD and culicids were used to assign scores. These criteria were applied uniformly in both the pre- and post-test evaluations. In the intervention stage, multimedia material was designed using Corel Draw, Publisher and PowerPoint viewer. For the interactive booklet, mosquito images were adapted from Cutwa and O’Meara [53], Centers for Disease Control and Prevention [54], and two images created with artificial intelligence were integrated. The production of the didactic material took into account the characteristics of web-based materials proposed by Prendes [55] and Prendes et al. [56], including organization of information (structure), motivational aspects, interactivity, multimedia, interface and navigation, usability, accessibility, and flexibility. For the implementation of the didactic strategy, the involvement of the community was fundamental, for this purpose, the participatory research workshop technique was established, which had the following stages: (a) training of the entire target population on the ecology of mosquitoes and VBD, emphasizing the problems of the study sector and to strengthen knowledge, the use of the iNaturalist application was implemented for the recognition of the diversity of mosquitoes; (b) section of dialogue with the community for identification and recognition of the problems (educational and environmental aspects on the health issue); (c) formulation of alternatives in the structure of the multimedia didactic material; and (d) socialization of the multimedia didactic material [57]. Finally, a final posttest evaluation was conducted, using the same instruments as the pretest [58], to identify the change in ecological and epistemological knowledge of the participants.

### 2.3. Data Analysis

For the evaluation of the impact of the educational strategy on social appropriation of knowledge regarding mosquito and VBD management, the data derived from the surveys (pretest and posttest) were initially analyzed through frequency tables and box plots. Subsequently, the change and improvement in knowledge before and after the educational intervention and the effects of variables such as gender, age, marital status, schooling, and occupation on these changes were analyzed. For this purpose, the Shapiro–Wilk normality test was performed to demonstrate the assumption of normality of the data, and when this was not met, a non-parametric test was performed. Next, the inference analysis of the data was carried out by means of the Wilcoxon rank test for related samples to demonstrate significant differences between the pretest and posttest results, in order to corroborate the efficiency of the didactic strategy and demonstrate the change in the ecological knowledge of mosquitoes and VBD. As a complement, a multinomial logistic regression was performed to determine the influence that gender, age, marital status, schooling, and occupation have on each person’s knowledge of VBD and mosquitoes. In this analysis, the post-test rating score obtained by each individual was used as a response variable. This choice allows us to evaluate how demographic characteristics influence the level of knowledge achieved after the intervention. The data obtained were organized in Excel and processed with the statistical package Jamovi version 2.5.6.

## 3. Results

### 3.1. Implementation of the Educational Intervention

**Figure 2:**
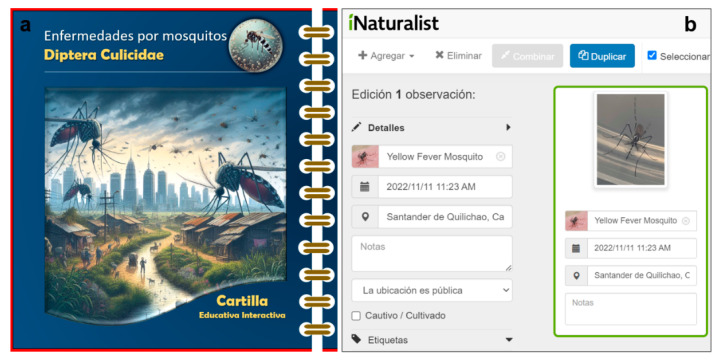
Educational intervention: (**a**) Cover of the interactive educational booklet and (**b**) use of iNaturalist to identify mosquitoes.

The multimedia didactic material (interactive booklet) was used, which included visual elements for a better understanding of the subject (Figure 2a, Appendix B). The booklet also included interfaces to strengthen the knowledge of the mosquito and its diseases, hyperlinks to learn more about the mosquito, the diseases it transmits, its symptoms, and recommended prevention actions. Additionally, the use of iNaturalist aided in the recognition of mosquito species in the region (Figure 2b). The participants were able to compare the photographs taken of the mosquitoes in their environment with the images available in the iNaturalist application. The visual features and detailed information of this application helped to improve participants’ ability to identify the morphology of mosquito species. In addition, the combination of the primer and the use of the iNaturalist promoted active participation and a more holistic approach to community health and education.

### 3.2. Pretest–Posttest Comparison

According to the findings derived from the application of the form (pretest), the group of people who intervened showed the following results: the sum of low knowledge (25%), medium knowledge (32%), and the sum of high knowledge (43%). The table of frequencies with respect to pretest and posttest knowledge shows changes in the level of knowledge of the participants (Table 1), showing a positive impact on the level of knowledge from very low to low, corresponding to 40 participants (23.3%), who changed their level of knowledge to medium (7.5%), high (7%) and very high (8.8%). Likewise, the three participants who had no knowledge changed to medium and high knowledge. In addition, 26.7% of the participants with medium knowledge in the pretest changed their level of knowledge to very high (*n* = 25) and high (*n* = 21). Similarly, 16.9% of the participants who obtained high knowledge in the pretest went on to have very high knowledge in the post-intervention test.

**Figure 3:**
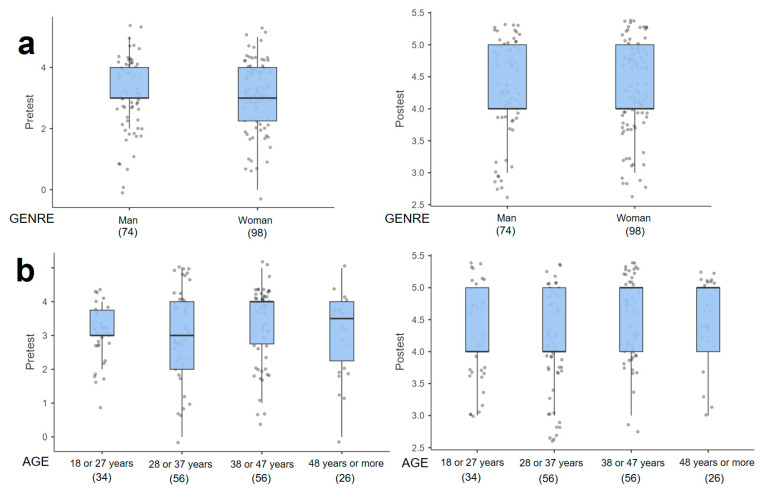
Box plots in relation to the level of knowledge reflected in the pretest and posttest grouped by socio-demographic variables: (**a**) Gender and (**b**) age.

**Figure 4:**
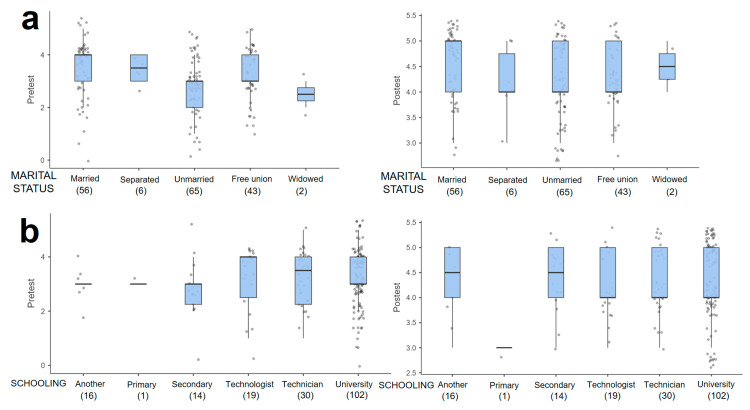
Box plots in relation to the level of knowledge reflected in the pretest and posttest grouped by socio-demographic variables: (**a**) Marital status and (**b**) schooling.

**Figure 5:**
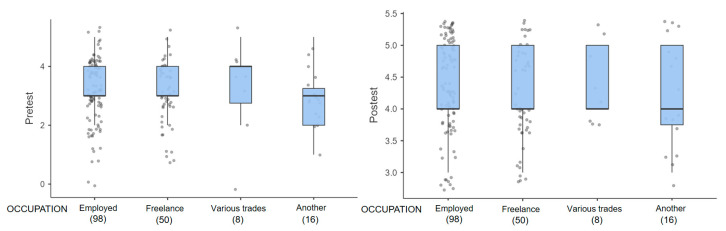
Box plots in relation to level of knowledge reflected in pretest and posttest grouped by socio-demographic variables: Occupation.

The sociodemographic variables of gender, age, schooling, marital status, and occupation in relation to the level of ecological and epistemological knowledge about mosquitoes and VBD presented a positive variation in the score with respect to the pretest and posttest. The variables gender and age presented a score that oscillates in the interquartile range of the box plot, between 2.0 and 4.0 in the pretest and for the posttest, a score between 4.0 and 5.0 is evident, corresponding to a high and very high knowledge (Figure 3a,b). The total range of scores by age group presents a greater dispersion of data in the pretest (Figure 3b). This dispersion is reduced in the posttest, demonstrating greater consistency of scores with higher levels of knowledge. The score for single people is observed to be more pronounced, progressing from a median of 2.5 in the pretest to 4.0 in the posttest (Figure 4a). This same effect is pre-presented in participants with a high school level of schooling (Figure 4b). The score for employees and independents tends to improve in a similar way (Figure 5).

The values of the difference between pretest and posttest did not meet the assumption of normality. The Wilcoxon rank test for related samples showed significant differences between pretest (*x* = 3.25) and posttest (*x* = 4.32) scores (*Z* = 94.5; *p* = < 0.001). The posttest scores were significantly higher than the pretest, which shows that the educational intervention in the community, through the multimedia didactic strategy with a citizen science approach, had a positive effect on the community’s ecological knowledge of VBD and mosquitoes.

Although the overall effect of the educational intervention had a positive impact on community ecological knowledge, the effect with respect to most of the sociodemographic variables on ecological knowledge, using multinomial logic regression, was not statistically significant. However, in participants who at the end had medium and high knowledge, the sociodemographic factor of marital status, independent of whether single or married, had a greater influence on ecological knowledge, and in turn, participants who were married or in union had a greater probability of acquiring high and very high knowledge (Table 2).

## 4. Discussion

Citizen science contributes in this sense since it involves the community in the process of generating and obtaining scientific knowledge [3]. At the same time, it encourages the participation of the population and facilitates the deepening of didactic processes, improving knowledge and awareness of environmental issues [59].

In terms of citizen science strategies, such as digital and technological innovation, multimedia, and interactive tools, the data analysis of this study revealed a positive influence towards high knowledge of the relationship between them and VBD in the majority of participants (*n* = 148). These findings are consistent with other similar studies where ecological knowledge has been improved in people participating in citizen science projects [5,6,18,20,21,38].

In addition, this study highlights how citizen science and its educational strategies had favorable results in learning processes in formal and non-formal education contexts [15,16,17,18,19,20]. Moreover, the technological tools used have influenced the interest and motivation of the participants in environmental and health issues [16].

Thus, the results of this study show for the first time the effectiveness of a multimedia didactic strategy with a citizen science approach to improving the understanding of VBD and the factors that influence its prevalence. It was possible to verify the improvement in the knowledge of the participants with very low, low, and medium knowledge who moved to the high knowledge ranges. This indicates that the educational intervention was more effective in those participants with greater knowledge and understanding needs, in addition to the motivation and capacity to develop and appropriate this topic of health interest. Hence, the importance of analyzing the motivational factors of each participant for this type of strategy, as concluded by Herodotou et al. [16] and Hamer et al. [28].

The variable schooling influences the final knowledge level of the participants, as shown in the box plot (Figure 4b). However, although the trend suggests that education is a relevant aspect of obtaining ecological knowledge [60,61], this influence was not statistically significant. The distribution of the data in the interquartile ranges of the sociodemographic groups versus the pretest and posttest, although showing improvements in the understanding of the topic, suggests no impact on the level of knowledge. This may be due to the fact that the participants do not have specific knowledge and training in ecological, entomological, or epidemiological factors. These sociodemographic variables do not have a significant influence on their level of ecological knowledge. Other studies are limited to describing the sociodemographic data and do not investigate their influence on knowledge [22].

However, the non-parametric statistical test of the Wilcoxon rank test revealed significant differences between the pretest and posttest, indicating that the posttest knowledge scores were significantly higher than those of the pretest, demonstrating a positive change in the participants’ knowledge of VBD and associated ecoepidemiological aspects. It is worth mentioning that the use of a pre- and posttest has been used in citizen science research to analyze independent and related population samples [16].

The multinomial logical regression presented an interesting result in reference to the marital status variable since it had a significant influence (*p* < 0.05) on the ecological knowledge of the target population. In particular, it was found that married or cohabiting participants were more likely to have high and very high knowledge than single participants. This could be due to the fact that married or cohabiting people tend to have a greater emotional stability and family responsibility that motivates them to be more receptive to the acquisition of ecological knowledge and health interests, with the purpose of structuring a safe and preventive environment in their family environment [62,63]. If there is a family with children, they visualize and propose the future welfare of their children, so they tend to implement strategies that promote sustainable development [64,65]. They are also more likely to be linked to community and EE networks that keep them informed and can accumulate more ecological knowledge [66]. This could explain why married or cohabiting people have the highest probability of acquiring a very high or high level of knowledge, compared to single people. In the case of single people, they tend to have more adventures and walks in the countryside, so they have a greater perception of the contemplation and enjoyment of the landscape and nature and, therefore, they could promote its care and conservation [67,68].

This last finding could be studied further by analyzing the variables that make marital status influence knowledge, such as the lifestyles and responsibilities of their family and work environment. As for the other sociodemographic variables, properly structured environmental education, providing accessible, inclusive, clear, and relevant information, can be more influential than the work context, age, gender, and schooling of individuals. This is consistent with studies where educational intervention can transform pro-environmental attitudes and behaviors and increase environmental awareness, overcoming the limitations imposed by sociodemographic factors, which minimizes the variability in the ecological knowledge acquired by participants [69,70].

In addition, the results of this study imply a promising contribution to community education and participation in environmental and health issues [22,57]. The integration of technological tools of citizen science to improve the understanding of environmental issues was also presented. However, it is important to consider in the methodological design, for future research, a control group to provide a baseline for comparing the results with the experimental group. This helps to determine whether the changes observed in the group that received the intervention are actually attributable to the intervention itself and not to other factors. The presence of a control group helps to control external variables that could influence the findings. In addition to evaluating the reduction in VBD cases in the educational intervention area. However, the lack of a systematic process for monitoring and updating health statistics in Santander de Quilichao highlights a significant institutional shortcoming. Compared to other departments, where more effective strategies have been implemented to manage VBD [71], the municipality faces a serious lack of coordination that compromises public health. It is imperative that stronger mechanisms be established to improve coordination between entities and to train technical personnel, thus ensuring an adequate response to local health needs.

## 5. Conclusions

The results of the present study suggest that the educational intervention strategy was effective in improving the knowledge level of the participants. The use of technological tools and multimedia didactic materials, with a citizen science approach, are strategies that should be used more frequently in NFE contexts. Citizen participation in the teaching–learning processes and the analysis of the influence of socio-demographic characteristics are fundamental to achieve a change in the ecological knowledge of the communities, which allows the appropriation of environmental knowledge that strengthens self-care and the prevention of VBD.

## Figures and Tables

**Table 1:** Frequencies of pre-test knowledge compared to posttest.

Pretest Knowledge	Frequencies	% of Total	Knowledge Posttest	Frequencies	% of Total
Very high knowledge	12	7.0%	Very high knowledge	10	5.8%
			High knowledge	2	1.2%
High knowledge	62	36.0%	Very high knowledge	**29**	**16.9%**
			High knowledge	32	18.6%
			Medium knowledge	1	0.6%
Medium knowledge	55	32.0%	Very high knowledge	**25**	**14.5%**
			High knowledge	**21**	**12.2%**
			Medium knowledge	9	5.2%
Low knowledge	29	16.9%	Very high knowledge	**13**	**7.6%**
			High knowledge	**7**	**4.1%**
			Medium knowledge	**9**	**5.2%**
Very low knowledge	11	6.4%	Very high knowledge	**2**	**1.2%**
			High knowledge	**5**	**2.9%**
			Medium knowledge	**4**	**2.3%**
No knowledge	3	1.7%	High knowledge	**2**	**1.2%**
			Medium knowledge	**1**	**0.6%**

**Table 2:** Coefficients of the multinomial logic regression model for sociodemographic variables and posttest knowledge.

Knowledge Posttest	Predictor	Estimator	EE	Z	*p*
Medium knowledge—High knowledge	Constant	−1.848	0.622	−29.721	0.003
	Marital Status:				
	Separated—Married	0.750	1.311	0.5724	0.567
	Unmarried—Married	1.465	0.706	20.745	0.038
	Free union—Married	0.279	0.792	0.3520	0.725
	Widowed—Married	−7.212	92.830	−0.0777	0.938
Very high knowledge—High knowledge	Constant	0.582	0.286	20.306	0.042
	Marital Status:				
	Separated—Married	−0.986	0.957	−10.310	0.303
	Unmarried—Married	−0.341	0.404	−0.8432	0.399
	Free union—Married	−1.121	0.442	−25.374	0.011
	Widowed—Married	−0.578	1.443	−0.4004	0.689

## Data Availability

All authors approve and certify the transparency of the data used. Further inquiries can be directed to the corresponding author.

## Appendix A

### Appendix A.1. Distribution of Participants across Different Sociodemographic Groups

**Variables**		***n***	**%**
Gender	Male	74	43.0
	Female	98	57.0
Age	18 to 27 years old	34	19.8
	28 to 37 years old	56	32.6
	38 to 47 years old	56	32.6
	48 years old or more	26	15.1
Marital status	Married	56	32.6
	Free union	43	25.0
	Unmarried	65	37.8
	Separated	6	3.5
	Widowed	2	1.2
Schooling	University	102	59.3
	Technologist	19	11.0
	Technician	30	17.4
	Secondary	14	8.1
	Primary	1	0.6
	Other	6	3.5
Occupation	Employed	98	57.0
	Freelance	50	29.1
	Various Trades	8	4.7
	Other	16	9.3

## References

[1] Fraisl D., Hager G., Bedessem B., Gold M., Hsing P.Y., Danielsen F., Hitchcock C.B., Hulbert J.M., Piera J., Spiers H. (2022). Citizen science in environmental and ecological sciences. Nat. Rev. Methods Primers.

[2] Jaeger J., Masselot C., Greshake Tzovaras B., Senabre Hidalgo E., Haklay M., Santolini M. (2023). An epistemology for democratic citizen science. R. Soc. Open Sci..

[3] Harrington E.G., Harrington E.G. (2019). Chapter 5—Citizen Science. Woodhead Publishing Series in Biomaterials.

[4] Callaghan C.T., Ozeroff I., Hitchcock C., Chandler M. (2020). Capitalizing on opportunistic citizen science data to monitor urban biodiversity: A multi-taxa framework. Biol. Conserv..

[5] Aristeidou M., Herodotou C., Ballard H.L., Higgins L., Johnson R.F., Miller A.E., Young A.N., Robinson L.D. (2021). How do young community and citizen science volunteers support scientific research on biodiversity? The case of inaturalist. Diversity.

[6] Hitchcock C., Sullivan J., O’Donnell K. (2021). Cultivating Bioliteracy, Biodiscovery, Data Literacy, and Ecological Monitoring in Undergraduate Courses with iNaturalist. Citizen Sci. Theory Pract..

[7] Holston J., Suazo H., Harris E., Coloma J. (2021). DengueChat: A social and software platform for community-based arbovirus vector control. Am. J. Trop. Med. Hyg..

[8] Pataki B.A., Garriga J., Eritja R., Palmer J.R., Bartumeus F., Csabai I. (2021). Deep learning identification for citizen science surveillance of tiger mosquitoes. Sci. Rep..

[9] Stevenson R., Merrill C., Burn P. (2021). Useful Biodiversity Data Were Obtained by Novice Observers Using iNaturalist During College Orientation Retreats. Citizen Sci. Theory Pract..

[10] Altrudi S. (2021). Connecting to nature through tech? The case of the iNaturalist app. Convergence.

[11] Mesaglio T., Callaghan C.T. (2021). An overview of the history, current contributions and future outlook of iNaturalist in Australia. Wildl. Res..

[12] Niemiller K.D.K., Davis M.A., Niemiller M.L. (2021). Addressing ‘biodiversity naivety’through project-based learning using iNaturalist. J. Nat. Conserv..

[13] Aristeidou M., Herodotou C., Ballard H.L., Young A.N., Miller A.E., Higgins L., Johnson R.F. (2021). Exploring the participation of young citizen scientists in scientific research: The case of iNaturalist. PLoS ONE.

[14] Sánchez-Clavijo L.M., Martínez-Callejas S.J., Acevedo-Charry O., Diaz-Pulido A., Gómez-Valencia B., Ocampo-Peñuela N., Ocampo D., Olaya-Rodríguez M.H., Rey-Velasco J.C., Soto-Vargas C. (2021). Differential reporting of biodiversity in two citizen science platforms during COVID-19 lockdown in Colombia. Biol. Conserv..

[15] Aristeidou M., Lorke J., Ismail N. (2022). Citizen science: Schoolteachers’ motivation, experiences, and recommendations. Int. J. Sci. Math. Educ..

[16] Herodotou C., Ismail N., Aristeidou M., Miller G., Lahnstein A.I.B., Khanaposhtani M.G., Robinson L.D., Ballard H.L. (2022). Online Community and Citizen Science supports environmental science learning by young people. Comput. Educ..

[17] López-Iñesta E., Queiruga-Dios M.Á., García-Costa D., Grimaldo F. (2022). Citizen science projects: An opportunity for education in scientific literacy and sustainability. Metode Sci. Stud. J..

[18] Stanković Z., Maksimović J., Osmanović J. (2018). Cognitive theories and paradigmatic research posts in the function of multimedia teaching and learning. Int. J. Cognit. Res. Sci. Eng. Educ..

[19] Kohler F., Kuthe A., Rochholz F., Siegmund A. (2022). Digital Education for Sustainable Development in Non-Formal Education in Germany and COVID-19-Induced Changes. Sustainability.

[20] Degner M., Moser S., Lewalter D. (2022). Digital media in institutional informal learning places: A systematic literature review. Comput. Educ. Open..

[21] Ku M.T., Chiou S.C., Chan H.T. (2020). Study of multimedia animation materials’design in environmental science education. J. Environ. Prot. Ecol..

[22] García M.A., Condo C.D.L., Quiñonez P., Alvarado J.R., De Gregorio S.A.F.G. (2021). Uso de TIC en la prevención del dengue asociado al proceso enseñanza-aprendizaje de enfermería clínica, Universidad de Guayaquil, Ecuador 2020. Bol. Malariología Salud Ambiental..

[23] Bedoya-Rodríguez F.J., Guevara-Fletcher C.E., Vera-Lizcano O. (2022). Identification, ecological indices and management of mosquitoes (Diptera: Culicidae) influencing environmental education processes in Colombian high schools. Int. J. Trop. Insect Sci..

[24] Ardoin N.M., Bowers A.W., Gaillard E. (2023). A systematic mixed studies review of civic engagement outcomes in environmental education. Environ. Educ. Res..

[25] Huang T.C., Chen C.C., Chou Y.W. (2016). Animating eco-education: To see, feel, and discover in an augmented reality-based experiential learning environment. Comput. Educ..

[26] Tao Y., Zhang G., Zhang D., Wang F., Zhou Y., Xu T. (2022). Exploring Persona Characteristics in Learning: A Review Study of Pedagogical Agents. Procedia Comput. Sci..

[27] Tomczyk Ł., Fedeli L., Tomczyk Ł., Fedeli L. (2022). Introduction—On the Need for Research on the Digital Literacy of Current and Future Teachers. Digital Literacy for Teachers.

[28] Hamer S.A., Curtis-Robles R., Hamer G.L. (2018). Contributions of citizen scientists to arthropod vector data in the age of digital epidemiology. Curr. Opin. Insect Sci..

[29] Dekramanjian B., Bartumeus F., Kampen H., Palmer J.R., Werner D., Pernat N. (2023). Demographic and motivational differences between participants in analog and digital citizen science projects for monitoring mosquitoes. Sci. Rep..

[30] Zhu H., Podesva P., Liu X., Zhang H., Teply T., Xu Y., Chang H., Qian A., Lei Y., Li Y. (2020). IoT PCR for pandemic disease detection and its spread monitoring. Sens. Actuators B.

[31] Kartini K., Sofia S., Nasrullah N. (2021). Development of Smartphone-Based Early Alerts and Mosquito Monitoring System and Geographic Instrument System Applications. Open Access Maced. J. Med. Sci..

[32] Polineni S., Shastri O., Bagchi A., Gnanakumar G., Rasamsetti S., Sundaravadivel P. (2022). MOSQUITO EDGE: An Edge-Intelligent Real-Time Mosquito Threat Prediction Using an IoT-Enabled Hardware System. Sensors.

[33] Barde P.V., Mishra N., Singh N. (2018). Timely diagnosis, use of information technology and mosquito control prevents dengue outbreaks: Experience from central India. J. Infect. Public Health.

[34] Carrillo M.A., Kroeger A., Sanchez R.C., Monsalve S.D., Runge-Ranzinger S. (2021). The use of mobile phones for the prevention and control of arboviral diseases: A scoping review. BMC Public Health.

[35] Andanaputra A.B.T., Ham H. (2021). Mobile Based Application of Mosquito Larvae Checking Reports: Malaka Sari Village Case. Procedia Comput. Sci..

[36] Fotakis E.A., Orfanos M., Kouleris T., Stamatelopoulos P., Tsiropoulos Z., Kampouraki A., Kioulos I., Mavridis K., Chaskopoulou A., Koliopoulos G. (2021). VectorMap-GR: A local scale operational management tool for entomological monitoring, to support vector control activities in Greece and the Mediterranean Basin. Curr. Res. Parasitol. Vector-Borne Dis..

[37] Purnama S., Susanna D., Achmadi U.F., Krianto T., Eryando T. (2021). Potential development of digital environmental surveillance system in dengue control: A qualitative study. Open Access Maced. J. Med. Sci..

[38] Pley C., Evans M., Lowe R., Montgomery H., Yacoub S. (2021). Digital and technological innovation in vector-borne disease surveillance to predict, detect, and control climate-driven outbreaks. Lancet Planet. Health.

[39] Abdulrahaman M.D., Faruk N., Oloyede A.A., Surajudeen-Bakinde N.T., Olawoyin L.A., Mejabi O.V., Imam-Fulanib Y.O., Fahmd A.O., Azeez A.L. (2020). Multimedia tools in the teaching and learning processes: A systematic review. Heliyon.

[40] Estallo E.L., Madelon M.I., Benítez E.M., Camacho-Rodríguez D., Martín M.E., Stewart-Ibarra A.M., Ludueña-Almeida F.F. (2024). Empowering Communities through Citizen Science: Dengue Prevention in Córdoba. Biology.

[41] Parra C., Cernuzzi L., Rojas R., Denis D., Rivas S., Paciello J., Coloma J., Holston J. (2020). Synergies between technology, participation, and citizen science in a community-based dengue prevention program. Am. Behav. Sci..

[42] Palmer J.R., Oltra A., Collantes F., Delgado J.A., Lucientes J., Delacour S., Bengoa M., Eritja R., Bartumeus F. (2017). Citizen science provides a reliable and scalable tool to track disease-carrying mosquitoes. Nat. Commun..

[43] Virgillito C., Longo E., De Marco C.M., Serini P., Zucchelli M.V., Montarsi F., Severini F., Rosà R., Da Re D., Filipponi F. (2024). Involving Citizen Scientists in Monitoring Arthropod Vectors of Human and Zoonotic Diseases: The Case of Mosquito Alert in Italy. Sci. Total Environ..

[44] Kosasih C.E., Lukman M., Solehati T., Mediani H.S. (2021). Effect of dengue hemorrhagic fever health education on knowledge and attitudes, in elementary school children in West Java, Indonesia. Ling. Cult. Rev..

[45] Hermida M.J., Santangelo A.P., Calero C.I., Goizueta C., Espinosa M., Sigman M. (2021). Learning-by-Teaching Approach Improves Dengue Knowledge in Children and Parents. Am. J. Trop. Med. Hyg..

[46] Bartumeus F., Oltra A., Palmer J.R. (2018). Citizen science: A gateway for innovation in disease-carrying mosquito management?. Trends Parasitol..

[47] Güler M.P.D., Afacan Ö. (2013). The impact of field trips on attitudes and behaviours related to sustainable environmental education. World Appl. Sci. J..

[48] Nuhoğlu H., İmamoğlu Y. (2018). An interdisciplinary nature education program for gifted primary school students and its effect on their environmental literacy. İlköğretim Online.

[49] Özalemdar L. (2021). The Effect on Environmental Attitude of the Active Learning Method Applied in Teaching the Biology Topic “Current Environmental Issues and Human” for 10th Grade Students. J. Turkish Sci. Educ..

[50] Chang S.J., Lee K.E., Yang E., Ryu H. (2022). Evaluating a theory-based intervention for improving eHealth literacy in older adults: A single group, pretest–posttest design. BMC Geriatr..

[51] Gobernación del Cauca (2019). Perfil Departamento del Cauca. Oficina Asesora de Planeación. Colombia. https://www.cauca.gov.co/Dependencias/OficinaAsesoradePlaneacion/InformacioneIndicadores/Perfil%20Departamento%20del%20Cauca.pdf.

[52] World Health Organization (2016). Knowledge, Attitudes and Practice Surveys: Zika Virus Disease and Potential Complications: Resource Pack.

[53] Centers for Disease Control and Prevention (2020). Life Cycle of Mosquitoes. Aedes Aegypti and Ae. Albopictus. https://www.cdc.gov/mosquitoes/pdfs/AedesLifeCycle_ESP-P.pdf.

[54] Cutwa M.M., O’Meara G.F. (2006). Photographic Guide to Common Mosquitoes of Florida.

[55] Prendes M.P. (2004). Diseño de cursos y materiales para teleenseñanza. Tecnol. Marcha.

[56] Prendes M.P., Martínez F., Gutiérrez I. (2008). Producción de material didáctico: Los objetos de aprendizaje. RIED. Rev. Iberoam. Educ. Distancia.

[57] Sánchez M.J., Fernández M., Diaz J.C. (2021). Técnicas e instrumentos de recolección de información: Análisis y procesamiento realizado por el investigador cualitativo. Rev. Cient. UISRAEL.

[58] Little T.D., Chang R., Gorrall B.K., Waggenspack L., Fukuda E., Allen P.J., Noam G.G. (2020). The retrospective pretest–posttest design redux: On its validity as an alternative to traditional pretest–posttest measurement. Int. J. Behav. Dev..

[59] Turrini T., Dörler D., Richter A., Heigl F., Bonn A. (2018). The threefold potential of environmental citizen science-Generating knowledge, creating learning opportunities and enabling civic participation. Biol. Conserv..

[60] Chaudhary M.G., Kabila V., Gupta A., Sailaja A., Garg R. (2023). The Role of Environmental Education in Promoting Environmental Conservation: A Systematic Review. Bol. Literatura Oral-The Literary J..

[61] Silva I.S., Cunha-Saraiva F., Ribeiro A.S., Bártolo A. (2023). Exploring the Acceptability of an Environmental Education Program for Youth in Rural Areas: ECOCIDADANIA Project. Educ. Sci..

[62] Dupont D.P. (2004). Do children matter? An examination of gender differences in environmental valuation. Ecol. Econ..

[63] Falla-Bermúdez T.V., León-Lesmes J. (2022). Manejo y control del dengue en familias de la comuna siete, Villavicencio, Meta. Boletín Semillero Investig. En Fam..

[64] UNICEF (2020). *Families, Family Policy and the Sustainable Development Goals*. Office of Research –Innocenti, Florence. www.unicef.org/innocenti/media/6226/file/UNICEF-Families-Family-Policy-and-SDGs-2020.pdf.

[65] Hosany A.S., Hosany S., He H. (2022). Children sustainable behaviour: A review and research agenda. J. Bus. Res..

[66] Movilla E.E.A., Becerra F.N.P. (2020). Redes comunitarias y de soporte social como recurso para el cuidado y el mantenimiento de la salud. Salud Soc. Uptc..

[67] Wells N.M., Lekies K.S. (2006). Nature and the life course: Pathways from childhood to adulthood. Child. Youth Environ..

[68] Evans G.W., Otto S., Kaiser F.G. (2018). Childhood origins of young adult environmental behavior. Psychol. Sci..

[69] Gifford R., Nilsson A. (2014). Personal and social factors that influence pro-environmental concern and behaviour: A review. Int. J. Psychol..

[70] Ortega-Sánchez D., Alonso-Centeno A., Corbí M. (2020). Socio-environmental problematic, end-purposes, and strategies relating to education for sustainable development (ESD) through the perspectives of Spanish secondary education trainee teachers. Sustainability.

[71] Hernández M., Arboleda D., Arce S., Benavides A., Tejada P.A., Ramírez S.V., Cubides Á. (2016). Metodología para la elaboración de canales endémicos y tendencia de la notificación del dengue, Valle del Cauca, Colombia, 2009–2013. Biomédica.

